# Retraction Note: Anti-diabetic, anti-oxidant and anti-hyperlipidemic activities of *Melastoma malabathricum* Linn. Leaves in streptozotocin induced diabetic rats

**DOI:** 10.1186/s12906-023-03996-9

**Published:** 2023-05-17

**Authors:** Vikas Kumar, Danish Ahmed, Pushpraj S Gupta, Firoz Anwar, Mohd Mujeeb

**Affiliations:** 1 Department of Pharmaceutical Sciences, Faculty of Health Sciences, Sam Higginbottom Institute of Agriculture, Technology & Sciences (SHIATS)-Deemed University, Allahabad, 211,007 Uttar Pradesh India; 2 Sidharatha Institute of Pharmacy, Dehradun, 248,001 Uttarakhand India; 3 Department of Photochemistry & Pharmacognosy, Faculty of Pharmacy, Jamia Hamdard, New Delhi, 110,062 India

**BMC Complementary Medicine and Therapies (2013) 13:222**.

10.1186/1472-6882-13-222

The Editor has retracted this article because of concerns with four of the figures, namely:

- Overlapping images in panels D and E in Fig. 23.
- Overlapping images in panels A and F in Fig. 25.
- Images in panels C,D, E and F in Fig. 18 are used in [1] which reports the results of a different experiment.
- Images in panels A and C in Fig. 24 and panels A, E and F in Fig. 25 are used in [2] which reports the results of a different experiment.

The Editor therefore no longer has confidence in the results and conclusions presented. Firoz Anwar disagrees with this retraction. Vikas Kumar, Danish Amar, Pushpraj S. Gupta and Mohd Mujeeb have not responded to correspondence from the Editor about this retraction.
